# Anomalous Case of Periodical Suspended Perception

**Published:** 1841-01-09

**Authors:** Joseph James Ridley


### ORIGINAL COMMUNICATION.

*Anomalous Case of Periodical Suspended Perception.* By JOSEPH JAMES RIDLEY, M. D.

To the Editors of the Medical Examiner.

The design of this paper is to present a concise statement of an interesting pathological phenomenon. I shall not attempt a philosophical dissertation on the rationale of its origin, progress, and spontaneous decline: I frankly confess that my inability satisfactorily to explain it has induced me to publish this memoir.

Harry, a man servant of Mrs. W., of N. C., was from his youth of a strong and vigorous constitution, plethoric habit, and in the intervals of his paroxysms of good general health. In about his thirty-fifth year, Doctor R. was requested to see him in a paroxysm simulating apoplexy. The remedies for apoplexy were used in vain. He lay perfectly torpid and insensible; the symptoms became hourly more threatening until recourse was had to cleansing the primæ viæ. The administration of a laxative restored him to sensation, and with the restoration of his perceptions all symptoms of disease disappeared. In a few days he had a second attack; the symptoms as before. The same treatment relieved him. His mistress, not famed for abundance of the "milk of human kindness," suspected his honesty, and cruelly scourged and burnt him in his paroxysms without making any impression upon his perceptions. His paroxysms periodically returned, and became more frequent gradually. He was likely to prove an expensive piece of property; and his mistress, resolved to incur no more expense, sold him at a reduced price to Dr. R., who, upon taking him home, essayed every power of the medical art to ascertain and remove the disease. He discovered that in his paroxysms the abdomen was uniformly extremely tense; the colon was, to all appearance, the seat of the disease. Upon making use of friction upon the abdomen, a vast quantity of flatus was eructated, and the urgent symptoms gradually gave way. After discharging an inconceivable quantity of gas, his senses returned; his paroxysms gradually became quotidian. Dr. R. was forced to take him with him whenever he was called from home, about the periodical recurrence of the paroxysm. The longer the paroxysm continued, the more difficult was it to restore him to sensibility. A most remarkable fact was the circumscribed spot upon which friction afforded relief; a five franc piece would cover the space on which it was necessary to rub; friction on any other part of the abdomen was certain to aggravate the symptoms fourfold. This spot was, I think, about four or six lines to the right of the umbilicus. A gentleman visiting at Dr. R.'s, upon one occasion endeavoured to relieve Harry in one of his paroxysms, having seen him speedily relieved by Dr. R. in so simple a manner. He was surprised and alarmed to see that, instead of affording relief, the man became every moment worse; and he hastened off after Dr. R., thinking that he was "in articulo mortis."

Another remarkable fact connected with this case, was the incredible force of his eructations: when properly rubbed, the flatus would escape with such force as to be heard one or two miles. I am aware, Messrs. Editors, that this seems rather a Munchausen story; it transcends the bounds of credibility, I know. It is difficult to believe a statement conflicting so palpably with the results of our observation. The lungs had no part in the production of the report; it was exclusively from the stomach and intestines. The statement, in its general outline, would be avouched by a whole neighbourhood, and several physicians, to whom the case was one of inexplicable difficulty and interest. When the paroxysm was coming on, before the power of locomotion was suspended, he would wander about, regardless of these things that affect the senses. Upon one occasion he was in the act of jumping into a well, and was most narrowly and opportunely rescued. Upon the return of sensibility, he was like a man suddenly aroused from sleep, before his perceptions are fully brought into exercise.

It is remarkable that neither diet, climate, labour, care, nor any thing else ordinarily connected with etyology of disease, had any influence over this case. His paroxysms uniformly came on independently of these things.

The disease lasted several years and finally exhausted itself. I think it probable that the proximate cause of the stupor was the pressure of the gas collected in the stomach, and colon upon the solar plexus, called by some physiologists the "brain of the stomach." As far as my investigations have extended, this case is without a parallel in pathology. If I had ever heard of gastro-enteric apoplexy, I should have some name for the disease. The symptoms in the paroxysm were clearly apoplectic; the full slow pulse and stertorous breathing, &c.

If by this paper, Messrs. Editors, a spirit of inquiry among the profession should be evoked, a solid benefit will have been done the cause of pathology. There are important facts evolved which, philosophically investigated, may throw much light upon vital phenomena. I commend the case to the profession and hope it may call out some reflections elucidating the difficulty; the points of difficulty that arise are, the periodicity of recurrence the stupor dependent upon gastric tension, the independence of the paroxysms of all the ordinary causes of disease; the necessity of applying friction to a circumscribed spot; the power of locomotion in the incipiency of the paroxysms remaining whilst perception was suspended; the force and loudness of the eructations, (much beyond the power of his lungs,) the final declension and disappearance of the disease, and, sirs, upon reflection, many other points of interest arise, challenging an elaborate investigation.

*Forsyth*, (*Geo.*) *Dec.* 12, 1840.

This is certainly a curious case. We do not remember to have met with any perfectly similar to it, although it is more nearly allied to hysterical and nervous disorders than to any others. These affections are in their nature extremely changeable, and their characters are rarely precisely alike. The view of the pathology of the disease given by our correspondent is probably the correct one, and the simple treatment of the disorder proves that there could have been little or no organic mischief. In cases of an analogous nature, we rely with great confidence of success upon injections of lac assafœtidæ, and half or an ounce of oil of turpentine. This remedy is by far the best in all cases which are classed under the term of nervous apoplexy; no revulsive is more powerful, and more perfectly harmless.

We agree with our correspondent that the case is highly interesting, and add our wishes to his that the recital of it may elicit others of a similar character, which would be the best means of throwing light upon it.—[Eds.]

